# CBP501 inhibits EGF-dependent cell migration, invasion and epithelial-to–mesenchymal transition of non-small cell lung cancer cells by blocking KRas to calmodulin binding

**DOI:** 10.18632/oncotarget.18598

**Published:** 2017-06-22

**Authors:** Naoya Saito, Naoki Mine, Donald W. Kufe, Daniel D. Von Hoff, Takumi Kawabe

**Affiliations:** ^1^ CanBas Co., Ltd., Numazu, Japan; ^2^ Dana-Farber Cancer Institute, Harvard School, Boston, MA, USA; ^3^ Translational Genomics Research Institute (TGen), Phoenix, AZ, USA

**Keywords:** CBP501, cell migration, invasion, EMT, calmodulin

## Abstract

The anti-cancer agent CBP501 binds to calmodulin (CaM). Recent studies showed that migration and metastasis are inhibited by several CaM antagonists. However, there is no available evidence that CBP501 has similar effects. Here we found that CBP501 inhibits migration of non-small cell lung cancer (NSCLC) cells *in vitro*, even in the presence of migration inducing factors such as WNT, IL-6, and several growth factors. CBP501 also inhibited epidermal growth factor (EGF) enhanced invasion and the epithelial-to-mesenchymal transition (EMT), and this inhibition was accompanied by (i) suppression of Akt and ERK1/2 phosphorylation, and (ii) suppression of expression of transcription factor Zeb1 and the mesenchymal marker Vimentin. A pull down analysis performed using sepharose-immobilized CaM showed that CBP501 blocks the interaction between CaM and KRas. Furthermore, EGF induced Akt activation and cell migration was effectively suppressed by KRas down-regulation in NSCLC cells. Stable knockdown of KRas also made cells insensitive to CBP501’s inhibition of growth factor-induced migration. Taken together, these results indicate that CBP501 inhibits binding of CaM with KRas and thereby suppresses the PI3K/AKT pathway, migration, invasion and EMT. These findings have identified a previously unrecognized effect of CBP501 on downstream KRas signaling mechanisms involving EMT and invasion, and provide support for the further clinical development of this agent.

## INTRODUCTION

Metastasis leads to death in about 90% of cancer patients [1]; accordingly, inhibition of cancer cell mobility would be of therapeutic benefit. Cell migration, invasion and EMT are the fundamental processes associated with cancer metastasis [2]. EMT contributes to cancer progression and is evoked during tumor invasion and metastasis [2-4]. EMT can be triggered by a variety of different molecules such as TGFβ, WNTs, IL-6, Notch, EGF, HGF, FGF, and HIF, and many diverse pathways have been implicated [2-4]. EMT is experimentally characterized by a decrease in the epithelial-state protein, E-cadherin, and increases of the mesenchymal-state proteins, vimentin [5].

Vimentin, one of the markers of EMT, is a regulator of cellular motility [6]. This was published in 2011, vimentin has gained attention as a potential molecular target for cancer therapy [7]. Overexpression of vimentin is associated with an increased capacity for cell migration and tissue invasion. Overexpression of vimentin was found to be an independent prognosticator for poor survival in resected NSCLC patients [8]. Others showed vimentin to be an Akt target, mediating cell motility and tissue invasion through its pathway [9].

The PI3K/Akt pathway is intimately connected to the migration of motile cells, including metastatic cancer cells [10]. It is possible that CaM-directed inhibitors exert their inhibition of cell migration via the PI3K/Akt pathway. W7, a CaM-directed inhibitor, suppresses EGF-induced Akt activation [11, 12]. Trifluoperazine, another CaM inhibitor, attenuates cancer cell invasion without cytotoxicity by suppressing Akt, and is a potential candidate for preventing cancer metastasis [13]. Thus, some CaM inhibitors might possibly serve as metastasis suppressors.

The Ras protein family (HRas, NRas, KRas4A and KRas4B) controls many cellular processes such as cell proliferation, transformation, differentiation, metastasis and apoptosis. Nussinov R *et al.* [14] proposed that CaM binding to KRas4B promotes cell proliferation and migration via the MAPK and Akt pathways. Wolfman *et al.* [15] proposed that cell migration is closely related to growth factor-dependent Akt activation and KRas/CaM interaction. In addition, McCormick *et al.* [16-20] proposed that blocking the specific interaction between KRas and CaM can be a novel approach to target KRas signaling in cancer therapy.

CBP501 is an anti-cancer agent that increases the sensitivity of tumor cells to platinum by CaM inhibition [21]. CaM inhibitors act by enhancing the antineoplastic effects of cisplatin [22]. Relative to other CaM inhibitors, CBP501 shows higher *in vitro* codrug activity with platinum [21]. Although several reports show that CaM inhibitors suppress cell migration and invasion, it was unknown whether CBP501 had such an inhibitory effects. Here, we positively established CBP501’s effects on cell migration, invasion and EMT and identified that the mechanism of CBP501’s inhibition of EGF-mediated cell migration and tissue invasion entailed reduced PI3K/Akt activation that ultimately stemmed from inhibition of KRas/CaM binding.

## RESULTS

### CBP501 inhibits NSCLC cell migration and invasion *in vitro*

Two mesenchymal-like NSCLC cell lines A549 (KRas mutation at codon 12) and H1299 (wild-type KRas and mutated NRas) were employed.

**Figure 1:**
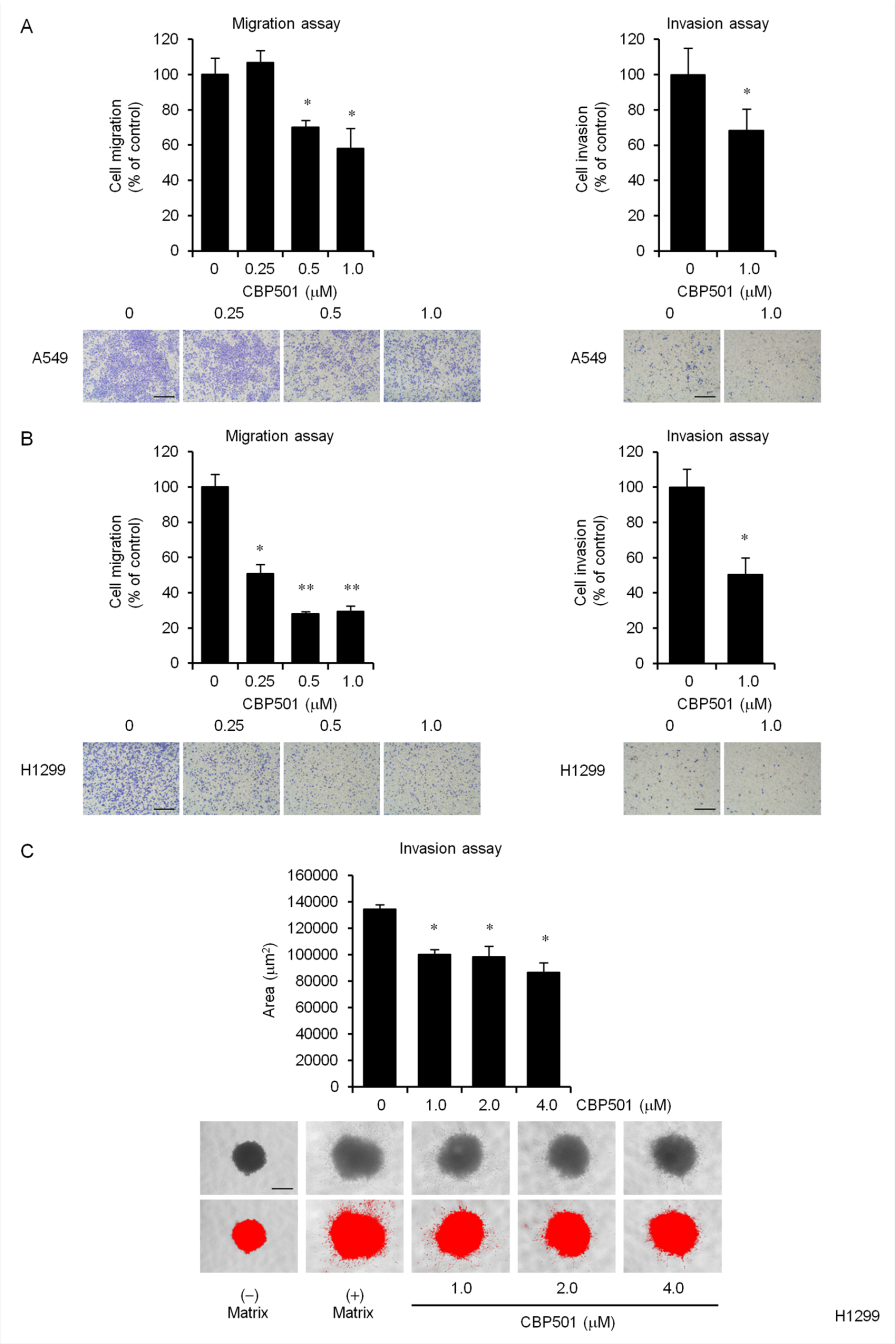
CBP501 prevents cell migration and invasion in A549 and H1299 cell lines Serum-starved A549 (**A**, left panel) or H1299 (**B**, left panel) cells were treated with CBP501 (0.25, 0.5 or 1 μM) for 24 h using a transwell assay. Serum-starved A549 (A, right panel) or H1299 (B, right panel) cells were treated with CBP501 (1 μM) for 48 h using matrigel invasion chamber. Cell migration and invasion %ages (above) were calculated from sample eluted from the membrane (*n* = 3). Photomicrographs of the observed cell migration (below). **(C)** Quantification of H1299 cell invasion in spheroid invasion assays. Cells were aggregated into spheroids and then induced to invade the surrounding matrix for 11 days. The total area of the invading spheroid was calculated with Image-J software and taken to be a measure of cell invasion (*n* = 3). Red signal threshold was set to capture the total structure. Scale bar is 500 μm. Data, the mean ± SD; * and **, *P* < 0.05 and *P* < 0.005, respectively.

The effect of CBP501 on migration was examined by a cell migration assay. As shown in Figure 1A and 1B left panels, 1 μM CBP501 inhibited 42% of total cell migration in A549 cells and 70% in H1299 cells. CBP501 also inhibited wound closure of A549 and H1299 in a scratch assay (Supplementary Figure 1A and 1B).

The effects of CBP501 on cell invasion were assessed by an *in vitro* matrigel cell invasion assay and a 3-D spheroid cell invasion assay. H1299 cells were found to be highly invasive whereas A549 were relatively lowly invasive (Supplementary Figure 2A and 2B). An *in vitro* invasion assay using BD BioCoat Matrigel invasion chamber showed that 1 μM CBP501 inhibited invasion by 32% in A549 and by 49% in H1299 (Figure 1A and 1B right panels). A 3-D spheroid cell invasion assay was performed to further investigate the effect of CBP501 on cell invasion in H1299 cells. The cells were grown as spheroids surrounded by an extracellular matrix (ECM) before inducing cell invasion by adding serum. Consistent with the transwell assay, CBP501 reduced the extent of spindle-like protrusions in the invasion matrix (Figure 1C). Analysis of these measurements at varying dose-levels indicated that CBP501 attenuates both cell migration and invasion in a dose-dependent manner.

To confirm that the inhibition of cell migration and invasion by CBP501 did not arise from cytotoxicity of CBP501, A549 and H1299 cells were analyzed for potential toxic effects in the presence of increasing concentrations of CBP501 using a WST8 cell viability assay. In these tests, cell viability was not affected by CBP501 at the 5 μM and 72 h (Supplementary Figure 3A and 3B). W7 and calmidazolium chloride (CMZ) had no effect on cell viability at concentrations up to 20 μM and 5 μM, respectively (Supplementary Figure 3C and 3D).

The CaM antagonists, trifluoperazine and ophiobolin A, had been found to prevent cell migration and invasion [13, 23]. Here, we confirmed that CaM antagonists (W7 and CMZ) also prevented cell migration (Supplementary Figure 4A; A549 and 4B; H1299) and prevented the formation of spindle-like protrusions in the invasion matrix (Supplementary Figure 5).

### EMT-inducing factors could not reverse CBP501-induced suppression of migration

**Figure 2:**
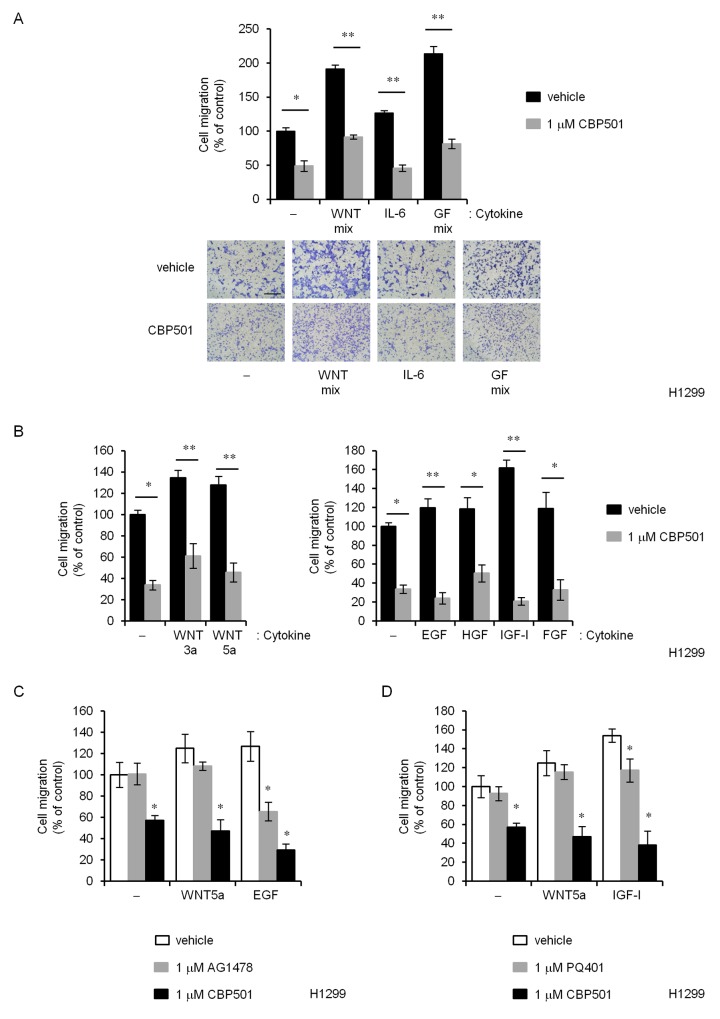
CBP501 prevents cell migration of H1299 cells by various EMT inducing factors **(A)** H1299 cells were treated over 72 h with CBP501(1 μM) in combination with various human (recombinant) migration-inducing factors; WNT mix (100 ng/mL WNT-3a and 100 ng/mL WNT-5a), 100 ng/mL IL-6, Growth Factor mix (GF mix; 500 ng/mL EGF, 50 ng/mL HGF, 200 ng/mL IGF-I and 100 ng/mL FGF). **(B)** (Left) H1299 cells were treated with WNT-3a or WNT-5a in combination with CBP501 for 72 h. (Right) H1299 cells were treated with EGF, HGF, IGF-I or FGF in combination with CBP501 for 72 h. **(C)** H1299 cells were treated with WNT-5a or EGF in combination with CBP501 or AG1478 for 72 h. **(D)** H1299 cells were treated with WNT-5a or IGF-I in combination with CBP501 or PQ401 for 72 h. Scale bar is 500 μm. Data, the mean ± SD; * and **, *P* < 0.05 and *P* < 0.005, respectively.

EMT induction can be triggered by a variety of factors including TGFβ, WNTs, IL-6, Notch, EGF, HGF, FGF, HIF, and many others [3]. A chemotaxis assay was performed with only H1299 cells because A549 did not survive for the required 48 h to 72 h in serum-free medium. CBP501-induced suppression of migration could not be reversed by any of the known EMT-inducers or inducer mixtures as shown in Figure 2A (WNT mixture, IL-6 and Growth factor mixture), Figure 2B (WNT3a, WNT5a, EGF, HGF, IGF-I and FGF), Figure 2C (WNT5a and EGF with CBP501 and AG1478) and Figure 2D (WNT5a and IGF-I with CBP501 and PQ401). In these experiments, EMT-inducing factors were added to the lower chambers of the migration test wells. Similar CBP501-induced suppression of migration was obtained with added TGFβ (Supplementary Figure 6A). AG1478, an EGFR inhibitor, specifically inhibited EGF-dependent migration but could not inhibit WNT5a-dependent migration. However, CBP501 inhibited cell migration in the presence of both WNT5a and EGF (Figure 2C). Similar results were obtained for an IGF-I inhibitor, PQ401 (Figure 2D). These findings suggest that CBP501 can inhibit cell migration even in the presence of a broad range of EMT-inducing factors.

### CBP501 reduces EMT-related proteins through inhibition of Akt pathway

EGF is known to induce EMT by promoting E-cadherin endocytosis [24], but it can also induce the expression of Zeb1, repressing E-cadherin transcription among other targets. Here we focused on the EGF-stimulated cell motility.

**Figure 3:**
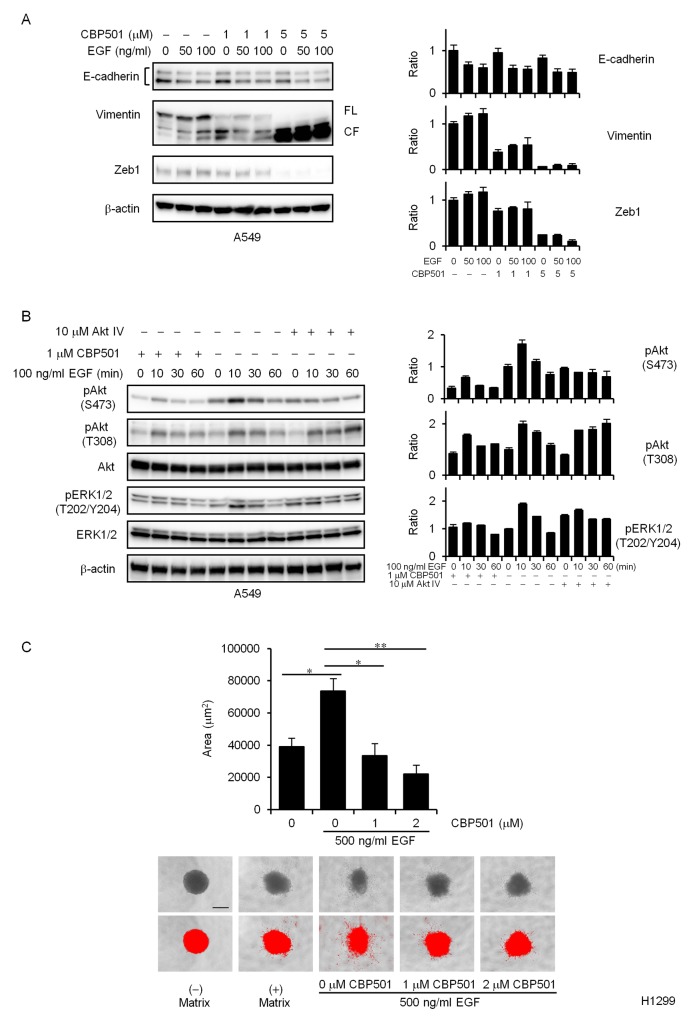
CBP501 attenuates EGF-dependent EMT via PI3K/Akt pathway **(A)** A549 cells were treated with or without EGF (50 or 100 ng/mL) in combination with CBP501 (1 or 5 μM) for 72 h. Cell lysates were analyzed by western blot assay with antibodies to E-cadherin, Vimentin, Zeb1, and β-actin. The measured band density was normalized relative to that of the control sample, with the control value set to 1. β-actin was used as a loading control. The results are the average relative intensity of three replicate blots. *FL*, full length; *CF*, cleaved fragment. **(B)** A549 cells were serum-starved for 1 day and then pretreated with CBP501 (1 μM) for 3 h before stimulating with EGF. The cells were treated with EGF (100 ng/ml) for 0-60 min. Cell lysates were analyzed by western blot assay (*n* = 3) with antibodies to pAkt, Akt, pERK1/2, ERK1/2, pSTAT3, STAT3 and β-actin. **(C)** Quantification of H1299 cell invasion by EGF stimulation in spheroid invasion assay. Cells were aggregated into spheroids and then induced to invade the surrounding matrix for 7 days with or without EGF (500 ng/mL) stimulation. The total area of each invading spheroid was calculated with Image-J software and taken to be a measure of cell invasion (*n* = 3). Red signal threshold was set to capture the total structure. Scale bar is 500 μm. Data, the mean ± SD; * and **, *P* < 0.05 and *P* < 0.005, respectively.

Western blot analysis demonstrated that expression of E-cadherin protein was significantly down-regulated by EGF stimulation, and that expression of vimentin and Zeb1 proteins was up-regulated in the EGF-treated cells compared to the untreated cells (Figure 3A). In contrast, 1 μM CBP501 reduced expression of EGF-induced Zeb1 and vimentin (Figure 3A). It is interesting to note that CBP501 might have induced vimentin proteolysis (Figure 3A, left panel) (*FL*, full length; *CF*, cleaved fragment). Similar effects were also seen upon treatment of A549 cells with CBP501 and TGFβ (Supplementary Figure 6B).

The PI3K/Akt pathway regulates cell migration and EMT [9, 10, 25]. Other CaM antagonists inhibit growth factor-induced migration and Akt activation [11, 12, 15]. BAPTA-AM, a cell-permeant Ca^2+^ chelator which has CBP501-like activity, was reported to suppress EGF-induced vimentin protein expression [26]. Moreover, Akt inhibition is known to induce caspase dependent vimentin proteolysis [9]. We therefore tested whether CBP501 represses EGF-induced Akt activation in A549 cells. As shown in Figure 3B, CBP501 significantly blocked the EGF-dependent Akt activation (pAkt, pERK1/2) in A549 cells. Akt IV inhibitor was used as a positive control of Akt inhibition. Similar effects were also seen in H1299 cells (data not shown). To further investigate the effect of CBP501 in EGF-dependent cell invasion, we performed 3-D spheroid cell invasion assay in H1299 cells. The spheroids were formed by ECM and cell invasion was induced by the addition of EGF. CBP501 reduced the formation of spindle-like protrusions upon EGF stimulation of cells in the invasion matrix (Figure 3C). These findings suggest that CBP501 inhibits EMT induction via the PI3K/Akt pathway.

### Knockdown of KRas by shRNA impairs the effects of CBP501 on inhibition of cell migration

**Figure 4:**
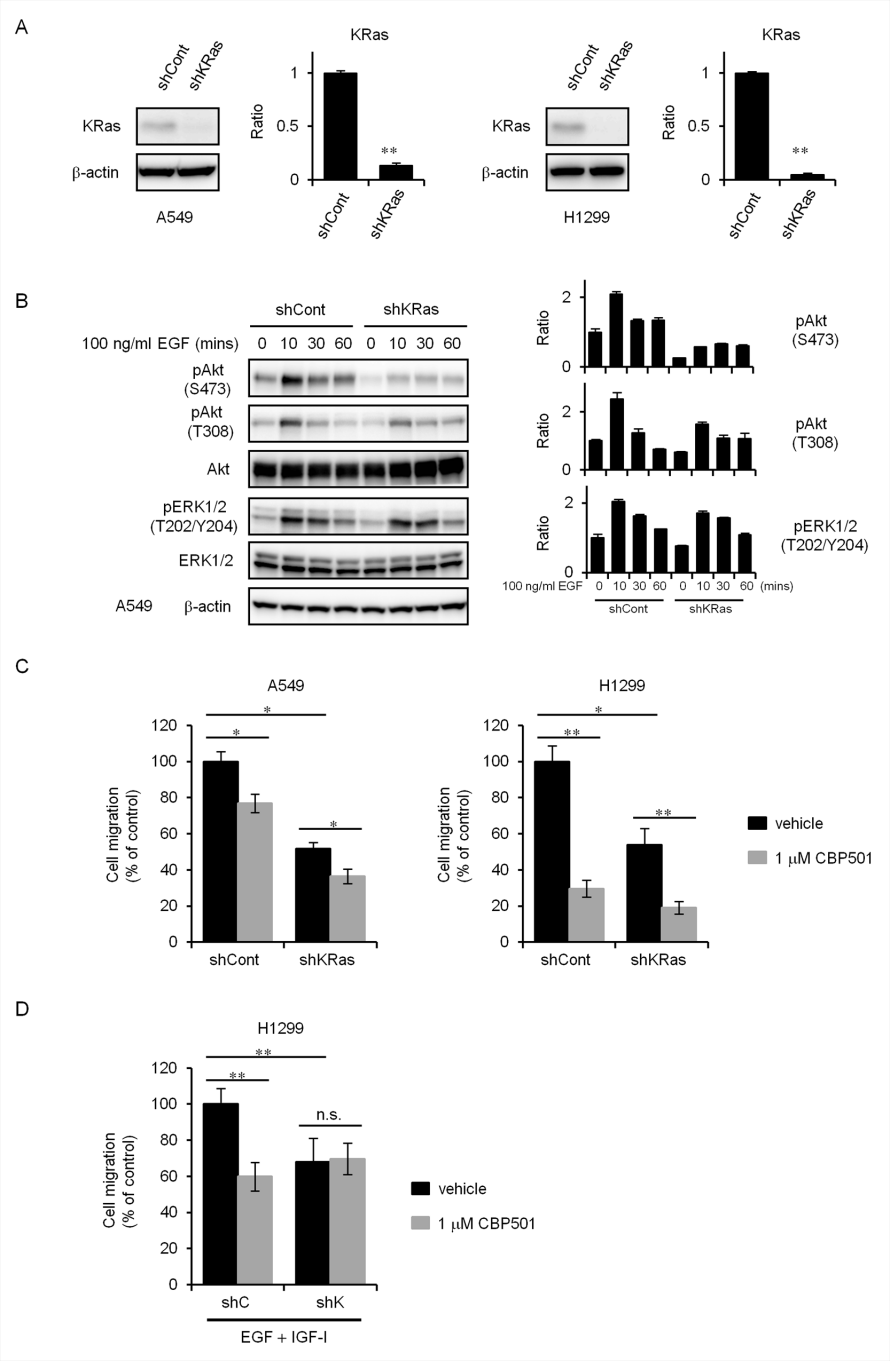
KRas knockdown cells attenuate inhibition of cell migration by CBP501 **(A)** Stable silencing of KRas in A549 and H1299 cells was assessed by comparing KRas expression in cell lysates from cells transduced with a control shRNA (shCont) or with KRas shRNA (shKRas) lentiviruses using a western blot assay. **(B)** Serum-starved A549 shCont and shKRas cells were treated with EGF (100 ng/mL) for 0-60 min. Cell lysates were analyzed by western blot assay (*n* = 3) with antibodies to pAkt, pERK1/2 and β-actin. **(C)** Serum-starved A549 or H1299 cells were treated with CBP501 for 24 h using transwell assay (*n* = 3). **(D)** Serum-starved H1299-shCont or H1299-shKRas cells were treated with EGF (500 ng/mL) and IGF-I (200 ng/mL) in combination with CBP501 for 72 h using transwell assay (*n* = 3). Data, the mean ± SD; * and **, *P* < 0.05 and *P* < 0.005, respectively; n.s., nonsignificant.

The Ras pathway is a major driver in lung adenocarcinoma [19]. KRas has long been a target for anti-cancer drug development, although many earlier strategies, including those targeting farnesyltransferase, have been disappointing. It was speculated in a review that a binding of CaM to KRas4B activates the PI3K/Akt pathway and that could enhance cell migration [14]. To confirm whether KRas relates to cell migration, we created a stable cell line exhibiting KRas knockdown by lentiviral-mediated shRNA interference in A549 and H1299 cells. The levels of KRas expression was analyzed by western blot analysis (Figure 4A).

KRas–deficient cells are reported to show decreased EGF-dependent Akt activation and cell migration [15, 27]. Concordantly, KRas-knockdown cells showed attenuated phosphorylation of Akt by EGF stimulation (Figure 4B). KRas-knockdown cells showed reduced migration with or without CBP501 compared with control cells of both the A549 and H1299 cell lines (Figure 4C). Interestingly, CBP501 did not suppress cell migration when the migration was induced by the mixture of EGF and IGF-I (Figure 4D) in the KRas-knockdown cell lines. These findings suggest that KRas contributes to the migration of NSCLC cells and is also at least partially involved in the inhibitory effect of CBP501 on cell migration.

### CBP501 inhibits CaM and KRas binding *in vitro*

**Figure 5:**
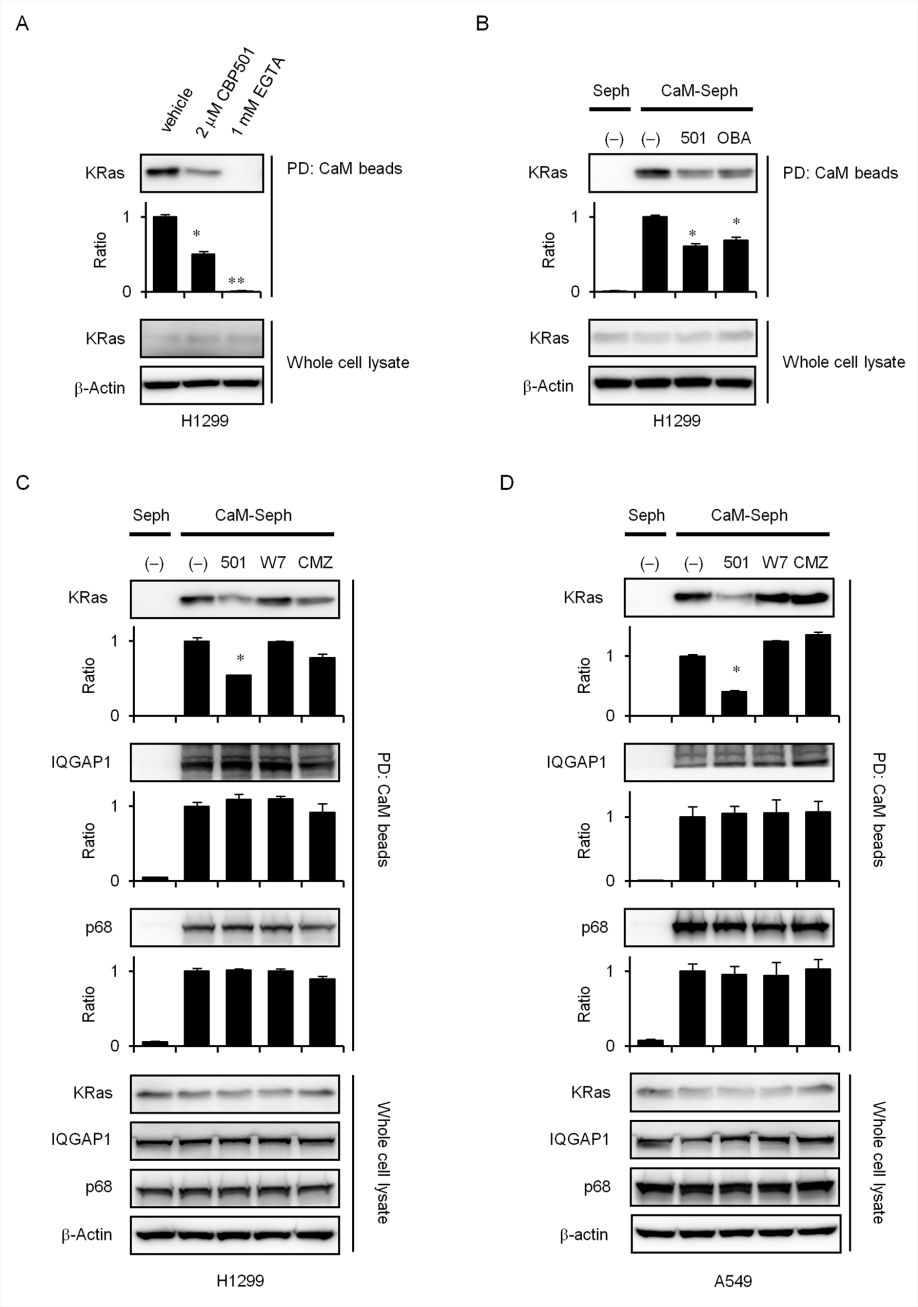
CBP501 inhibits KRas/CaM interaction **(A)** Cellular lysates (1 mL) from H1299 cells were incubated with CaM-sepharose together with CBP501 (2 μM) or EGTA (1 mM) in the presence of Ca^2+^ for 2 h as indicated in Materials and Methods. The presence of KRas and β-actin were analyzed by western blot assay using specific antibodies. **(B)** Cellular lysates (1 mL) from H1299 cells were incubated with CaM-sepharose (CaM-seph) or with Plain (Seph) beads together with CBP501 (2 μM) or OBA (2 μM) in the presence of Ca^2+^ as indicated in Materials and Methods. Cellular lysates (1 mL) from H1299 cells **(C)** and A549 cells **(D)** were incubated with CaM-sepharose (CaM-seph) or with Plain (Seph) beads together with CBP501 (2 μM), W7 (20 μM) or CMZ (5 μM) in the presence of Ca^2+^ as indicated in Materials and Methods. Cell lysates were analyzed by western blot assay with antibodies to KRas, IQGAP1, p68 and β-actin. The results are the average relative intensity of three replicate blots.

Recent evidence suggests that Ca^2+^-CaM selectively modulates KRas4B signaling [14, 28-30]. Unlike other isoforms, KRas4B can interact with CaM in a Ca^2+^-dependent manner [15, 31-33]. McCormick *et al.* recently proposed that blocking specific interactions between CaM and KRas may provide a novel approach to target KRas signaling in cancer [16-20]. Correspondingly, the highly potent CaM inhibitor ophiobolin A interferes with KRas activity by binding to and inhibiting CaM directly, as observed through reduced mammosphere formation [34]. The interaction between CaM and KRas was reported to regulate growth factor-dependent Akt activation and cell migration [15]. To investigate whether CBP501 inhibits CaM and KRas binding, we analyzed this interaction *in vitro* using CaM-sepharose pull down assays with H1299 cell lysate. KRas was able to bind to CaM-sepharose in the presence of Ca^2+^, and the binding could be suppressed by added CBP501 and EGTA (Figure 5A). As shown in Figure 5B and 5C, CBP501, Ophiobolin A (OBA) and CMZ specifically suppressed CaM/KRas binding, but did not inhibit interaction of CaM with either IQGAP1 [35] or p68 RNA helicase [36], two other CaM-binding proteins. CaM/KRas binding inhibition by CBP501 was also seen with A549 cell lysate (Figure 5D). These results using three different CaM antagonists suggest that CBP501 is quite specific at suppressing CaM/KRas binding.

## DISCUSSION

In the present study, we investigated mechanisms by which CBP501 inhibits cell migration, invasion and EMT. The seminal findings of this work are: (i) CBP501 inhibits NSCLC cell migration and invasion *in vitro*; (ii) many EMT-inducing factors can not reverse the suppression of migration by CBP501; (iii) CBP501 reduces expression of EMT-induced proteins with inhibition of the Akt phosphorylation;(iv) knockdown of KRas by shRNA negates inhibition of migration by CBP501; and (v) CBP501 inhibits the formation of a complex between CaM and KRas. These findings indicate that CBP501 inhibits EGF-dependent cell migration by inhibiting the formation of CaM/KRas complex and suppressing Akt activation.

**Figure 6:**
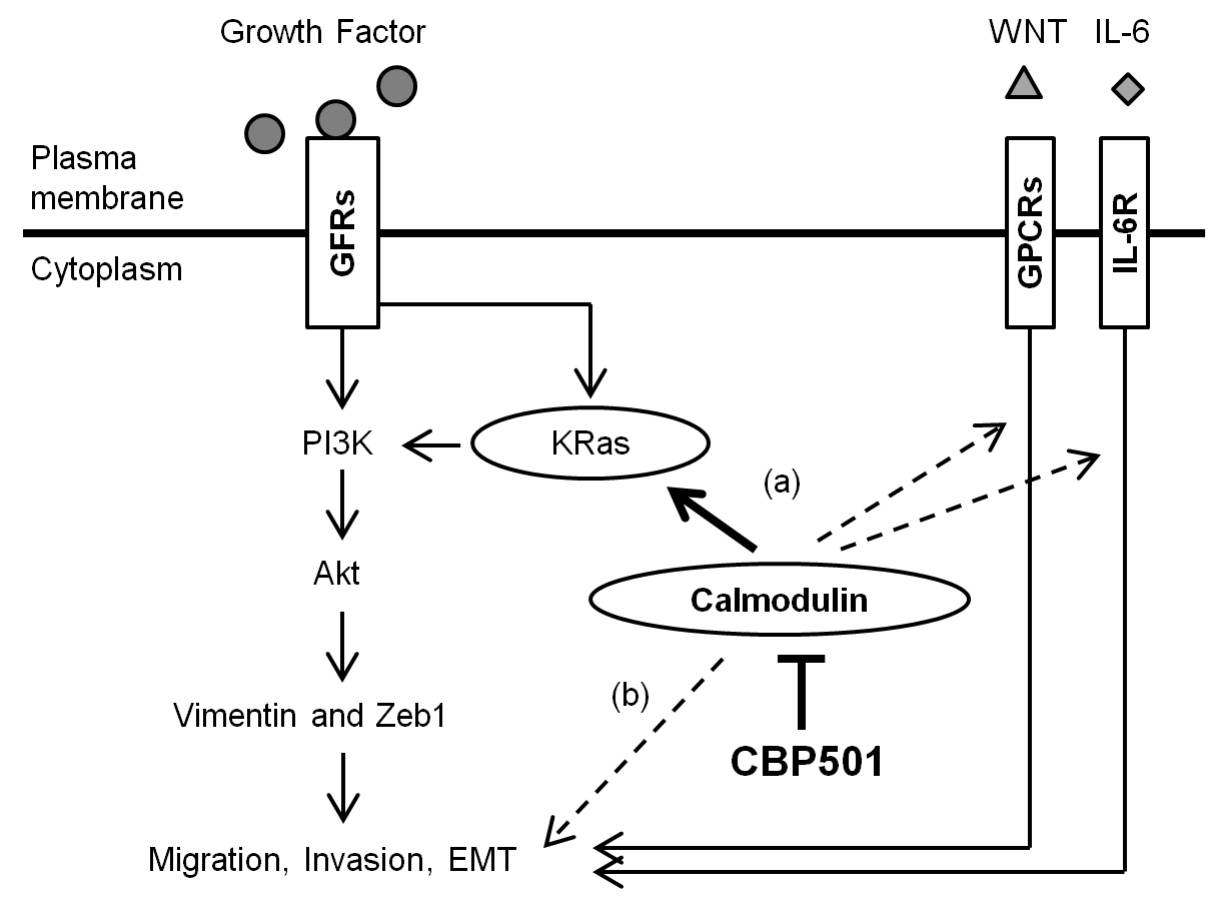
Schematic representation that summarizes the proposed mechanism of action of CBP501 as described in the text CBP501 interacts with CaM, blocking its observed (or potential) interactions with KRas, GPCRs or IL-6R, thereby affecting a variety of pathways related to cell migration, invasion and EMT. GFRs, growth factor receptors; GPCRs, G protein-coupled receptors; IL-6R, interleukin-6 receptor.

Since CaM is involved in many cellular phenomena, it requires much effort to clarify the complete function of any CaM-directed inhibitor [37]. Given CBP501’s observed anti-CaM activity, CBP501 might conceivably have affected CaM’s action by two avenues: (a) by inhibiting CaM-dependent signaling pathways that control cell migration; (b) by inhibiting CaM-dependent cytoskeletal processes including actin filament formation and the action of microtubule motor proteins such as dynein and kinesin (Figure 6). In the current work, we clarified that the CaM-directed inhibitor CBP501 regulates EGF-dependent cell migration by inhibiting CaM/KRas binding and through the Akt pathway (Figure 6). CBP501 can inhibit cell migration in the presence of a broad-range of EMT-inducing factors other than EGF. Future analysis is necessary to investigate the basis for this broad inhibition of cell migration caused by mechanisms other than EGF stimulation.

EMT is regulated by several signaling pathways that interact to produce a full range of EMT responses [38]. EMT induction is increased through crosstalk and cooperation between these distinct pathways. WNT signaling can promote EMT by inhibiting glycogen synthase kinase-3β (GSK3β) to stabilize β-catenin. The CaM antagonist trifluoperazine suppresses tumor spheroid formation and down-regulates WNT/β-catenin signaling [39]. Interleukin-6 (IL-6) is a cytokine present in tumor microenvironment and can promote EMT through enhancing STAT3, MAPK and Akt signaling [40].

Ca^2+^ and CaM have been reported to participate in cell migration [41]. Ca^2+^-CaM was found to inhibit the binding of F-actin and Cdc42 to IQGAP1 [42, 43]. IQGAP1 acts as a scaffold protein that couples cell signaling to processes involving the actin and microtubule cytoskeletons during cell migration [44]. Binding between Ca^2+^-CaM and IQGAP1 was found to attenuate cell migration [45]. Interaction between CaM and IQGAP1 was also found to reduce induction of ERK activity by EGF [46]. As shown in Figure 3B, EGF-induced ERK activity was reduced by CBP501. Thus, CBP501 may inhibit not only the PI3K/Akt pathway, but also the Raf/MEK/ERK pathway. Cdc42 is another possible site of CaM action that may be affected by CBP501. It is a member of the Rho family of small GTPases and has been shown to induce filopodia formation, a process entailing cytoskeleton organization and consequently possibly affecting cell migration [42]. Initial results from the CaM-sepharose pull-down experiments indicated that CBP501 might also possibly enhance CaM/Cdc42 binding (data not shown). A further possible site of CaM action that might have been affected by CBP501 was the interaction between Ca^2+^-CaM and p68 RNA helicase, which was reported to promote cell migration and metastasis [36]. The p68 interacts with microtubules in the presence of Ca^2+^-CaM and can function as a microtubule motor. The IQ-like motif peptide of p68 interrupts the interaction between p68 and Ca^2+^-CaM and inhibits cell migration [36]. Since CBP501 did not inhibit the binding between CaM and p68, CBP501 is unlikely to be related to this mechanism (Figure 5C and 5D). Further analysis is necessary for these migration mechanisms.

The PI3K/Akt pathway regulates cell migration [10]. We tested whether Akt inhibitor Akt IV inhibits cell invasion with transwell. Akt IV inhibited invasion both A549 and H1299 cells (Supplementary Figure 7A). CaM directly also binds to Akt and modulates Akt translocation to the plasma membrane [47, 48]. We tested whether CBP501 inhibits binding of Akt/CaM using pull down assay. CBP501 did not inhibit Akt/CaM interaction (Supplementary Figure 7B). Furthermore, CaM is an integral component of a K-Ras4B/PI3Kɑ ternary complex [49]. CBP501 may interfere with the trimer formation.

Given that the interaction between KRas and CaM was found to be significant here, there are three different regions in the KRas protein that are known to be important for its interaction with CaM [31]. These are the hypervariable region, the ɑ-helix between amino acids 151 and 166, and the Switch II region [31, 33]. Further investigation is needed to identify whether there is direct interaction of CBP501 with any of these sites on KRas. The selective inhibition of KRas-CaM binding by CBP501 may instead result from a stable, KRas-incompatible conformer assumed by CaM in a stable CaM-CBP501 complex or from CBP501’s blocking of CaM’s usual sites of interaction with KRas in such a CaM-CBP501 complex.

In conclusion, this study provides the first evidence that CBP501 can inhibit lung cancer cell migration, invasion and EMT. In the case of EGF induced migration, invasion and EMT it was shown that CBP501 inhibited EGF-induced PI3K/Akt pathway via suppression of CaM/KRas binding. CBP501 provides a novel approach to target KRas signaling in cancer in addition to the known anti-cancer action which is the augmentation of cytotoxicity of platinum agents to tumor cells [21].

## MATERIALS AND METHODS

### Cell lines and reagents

Human NSCLC cell lines, A549 (KRas mutant) and H1299 (KRas wild-type) were purchased from American Type Culture Collection (ATCC, Manassas, VA). Cell lines were authenticated by short tandem repeat analysis. Cell lines were initially grown and low passage stocks were cryopreserved in liquid nitrogen. Cells were replaced by frozen stocks after 3 months of continuous culture. A549 cells were cultured in Ham’s F-12K (Kaighn’s) medium (Gibco; Thermo Fisher Scientific Inc., Waltham, MA) containing 10% fetal bovine serum (FBS) (Gibco) and 1% penicillin/streptomycin (PS) (Gibco). H1299 cells were cultured in RPMI1640 medium (Sigma-Aldrich, St. Louis, MO) containing 10% FBS, 1% PS. Cells were grown at 37°C in humidified atmosphere consisting of 5% CO_2_ and 95% air. CBP501 was manufactured by Lonza (Braine-l’Alleud, Belgium). Akt inhibitor IV, EGFR inhibitor AG1478 and IGF-IR inhibitor PQ401 were purchased from Millipore (Bellerica, MA). Ophiobolin A was purchased from Enzo life science (Lausen, Switzerland). CaM inhibitor, W7 and Calmidazolium chloride were purchased from Sigma-Aldrich. Recombinant human WNT-3a, WNT-5a, IL-6, EGF, HGF, IGF-I and FGF were purchased from R & D systems (Minneapolis, MN).

### *In vitro* transwell migration assay

Cell migration assays were performed in 24-well plates with 8 μm pore sized transwell chambers (Corning, #3422, Kennebunk, ME). The cells were serum-starved overnight in serum-free medium (0.1% BSA). A total 1-5 x 10^4^ cells in serum-free medium (0.1% BSA) were added to the upper chamber. RPMI1640 (2% FBS) or F12K (10% FBS) were added to the lower chamber. A549 and H1299 cells were cultured for 24 h to observe migration. The migrated cells were stained with crystal violet (BD biosciences, San Jose, CA) for 15 min at room temperature. The crystal violet stain was then removed from the chambers, and cells were washed thrice with distilled water (dH_2_O). Cells on the upper chamber of the membrane were scraped off using a cotton swab. The experiment was performed in triplicates for all conditions described. Cell migrations were captured using an Olympus CKX41 microscope (Olympus Optical Co., Hamburg, Germany) via Moticam 580 digital camera (Motic, Hong-Kong, China) at 40-fold magnification. Crystal violet was then eluted with extraction buffer (2.5% methanol, 2.5% Isopropanol, 30% ethanol and 0.5% acetic acid) in dH_2_O and the eluted samples were transferred to a 96-well plate. The migrated stained cells were quantified by colorimetric measurement at 550 nm. The absorbance at 550 nm (OD_550_) was measured using a Sunrise basic microplate reader (Tecan Austria GmbH), in accordance with the manufacturer’s instructions.

### *In vitro* transwell matrigel invasion assay

Cell invasion assays were performed with a BioCoat Matrigel Invasion Chamber (Corning, Bedford, MA). The cells were serum-starved overnight in serum-free medium (0.1% BSA). A total of 2 x 10^4^ cells in serum-free medium (0.1% BSA) were added to the top invasion chambers of 24-well transwell plates containing a cell culture insert with an 8 μm pore size. RPMI1640 (2% FBS) or F12K (10% FBS) were added to the lower chamber. A549 and H1299 cells were cultured in the culture insert to observe the invasion for 48 h. The procedure after the crystal violet staining is the same as for the cell migration assay.

### 3-D Spheroid cell invasion assay

The invasive ability of the H1299 cells was investigated using the Cultrex 96 Well 3-D Spheroid BME Cell Invasion Assay according to the manufacturer’s instructions (Trevigen, Inc., Gaithersburg, MD). Briefly, 250 cells were resuspended for spheroid formation in ECM solution and gently pelleted in a 96 well round bottom spheroid formation plate. After 3 days, spheroids were imaged. To induce invasion, the invasion matrix and a serum-containing medium were added to each well. Cells invaded the surrounding matrix for 7 to 11 days. Images were captured using an Olympus CKX41 microscope via Moticam 580 digital camera at 40-fold magnification. The area of each cell mass (pre- and post-invasion) was measured using Image-J software (NIH, Bethesda, MD). The difference between the pre- and post-invasion areas was used a measure of cell invasion.

### Chemotaxis assay

The chemotaxis assay was performed using transwell chambers. The cells were serum-starved overnight in serum-free medium (0.1% BSA). Briefly, RPMI1640 (0.5% FBS) containing the chemoattractant (eg, EGF, IGF-I, etc.) was added to the lower chamber. A total of 1 × 10^4^ cells in serum-free medium (0.1% BSA) were added to the upper chamber. Then, the chamber was incubated at 37°C for 72 h. The procedure after the crystal violet staining is the same as for the cell migration assay.

### Cell extracts and Western blot assay

To prepare extracts from the cell culture, cells were washed with PBS and resuspended in lysis buffer [20 mM Tris-HCl (pH 7.5), 150 mM NaCl, 1 mM EDTA, 1 mM EGTA, 0.5% (w/v) Nonidet P40, Complete Protease Inhibitor Cocktail (Roche, Basel, Switzerland), PhosSTOP Phosphatase Inhibitor Cocktail (Roche)]. Cells were vortexed with periodic cooling on ice for 1 h and then centrifuged at 13,000 rpm for 10 min at 4°C. Cell extracts were resuspended in a loading buffer and stored at -80°C until analysis by Western blotting. Proteins were separated by electrophoresis on the Mini-protean TGX gel (Bio-Rad, Hercules, CA) and transferred to Immobilon-P polyvinylidene difluoride (PVDF) membranes (Millipore, Bedford, MA). Antibodies against Vimentin (V9), K-Ras (F234), IQGAP1 (H109), p68 RNA Helicase (H144) or β-actin (C4) were purchased from Santa Cruz Biotechnology (Santa Cruz, CA). Antibodies against Zeb1 (3396), phospho-Akt (Ser473) (4060), Akt (Pan) (4691), phospho-Erk1/2 (Thr202/Tyr204) (4370), Erk1/2 (4695), phospho-Stat3 (Tyr705) (9145), Stat3 (9132) and rabbit IgG, as well as mouse IgG conjugated with horseradish peroxidase (HRP) were purchased from Cell Signaling Technology (Danvers, MA). Antibody against E-cadherin (610182) was purchased from BD Biosciences (San Jose, CA).

### Establishment of stable K-Ras silenced NSCLC cells

K-Ras shRNA lentiviral particles (sc-35731-v) and control shRNA lentiviral particles (sc-108080) were purchased from Santa Cruz Biotechnology. To establish stable knockdown cell lines, NSCLC cells were transfected with shRNA lentiviral particles, and cultured in the presence of 2 μg/mL puromycin. Cells were characterized for the K-Ras expression level by western blot assay.

### Pull down assay with CaM-sepharose

The pull-down assay was performed using CaM-Sepharose Beads (BioVision, Milpitas, CA). Cells were lysed with pull-down buffer [50 mM Tris-HCL (pH7.5), 150 mM NaCl, 1 % Triton X-100, 1 mM dithiothreitol (DTT), Complete Protease Inhibitor Cocktail and PhosSTOP Phosphatase Inhibitor Cocktail]. Clear lysate was collected after centrifugation at 13,000 rpm for 10 min at 4 °C. CaM-sepharose beads (Biovision, Milpitas, CA) and Plain beads (Sepharose 6 fast flow, GE healthcare, Uppsala, Sweden) were equilibrated with pull-down buffer. CaM-sepharose beads were pretreated with CBP501 or other compounds for 1 h at 4 °C. The clarified cell lysates were incubated with CaM beads or Plain beads in combination with CBP501 or other compounds for 2 h at 4 °C in the presence of 0.1 mM CaCl_2_. After incubation, beads were washed thrice with pull-down buffer. The proteins bound to the beads were eluted by boiling for 5 min in 5 × SDS-PAGE loading buffer. Then the samples were analyzed by Western blotting.

### Statistical analysis

Data were analyzed using the *t* test (Student’s *t* test or Welch’s *t* test). All experiments were performed in triplicate. Data are expressed as the mean ± standard deviation (SD). A *P* value of < 0.05 or < 0.005 was considered significant.

## SUPPLEMENTARY MATERIALS FIGURES


