# Supplementary material for: CBP501 inhibits EGF-dependent cell migration, invasion and epithelial-to–mesenchymal transition of non-small cell lung cancer cells by blocking KRas to calmodulin binding
Source: Oncotarget. 2017 Jun 22;8(43):74006–18. doi: 10.18632/oncotarget.18598 (PMC5650319; doi:10.18632/oncotarget.18598)
Supplement: Supplementary file 1 [file oncotarget-08-74006-s001.pdf]

CBP501 inhibits EGF-dependent cell migration, invasion and epithelial-to–mesenchymal transition of non-small cell lung cancer cells by blocking KRas to calmodulin binding

SUPPLEMENTARY MATERIALS

Wound healing assay

The wound healing assays was performed using culture-inserts (ibidi GmbH, Martinsried, Germany). 1 × 10^4 cells of each NSCLC cell line were plated in 35-mm dishes with culture-inserts. When cells grew to confluence, inserts were then removed with sterile forcepts to create a wound field of approximately 400 µm. After removing the cellular debris with PBS, cells were exposed to CBP501 (1 µM and 2µM) for 17-48 h. Images were captured using an Olympus CKX41 microscope via Moticam 580 digital camera at 40-fold magnification.

Cell viability assay

Cells (5 × 10^3) were seeded on 96-well plates and incubated for 24 h. After incubation with CBP501, W7 or CMZ, cell viability was analyzed with a cell counting kit-8 (Dojindo, Kumamoto, Japan). Reagent solution was added to each well, the 96-well plates were incubated for 3 h at 37°C, and the absorbance at 450 nm (OD_{450}) was measured using a Sunrise basic microplate reader (Tecan Austria GmbH, Grödig, Austria), in accordance with the manufacturer's instructions.

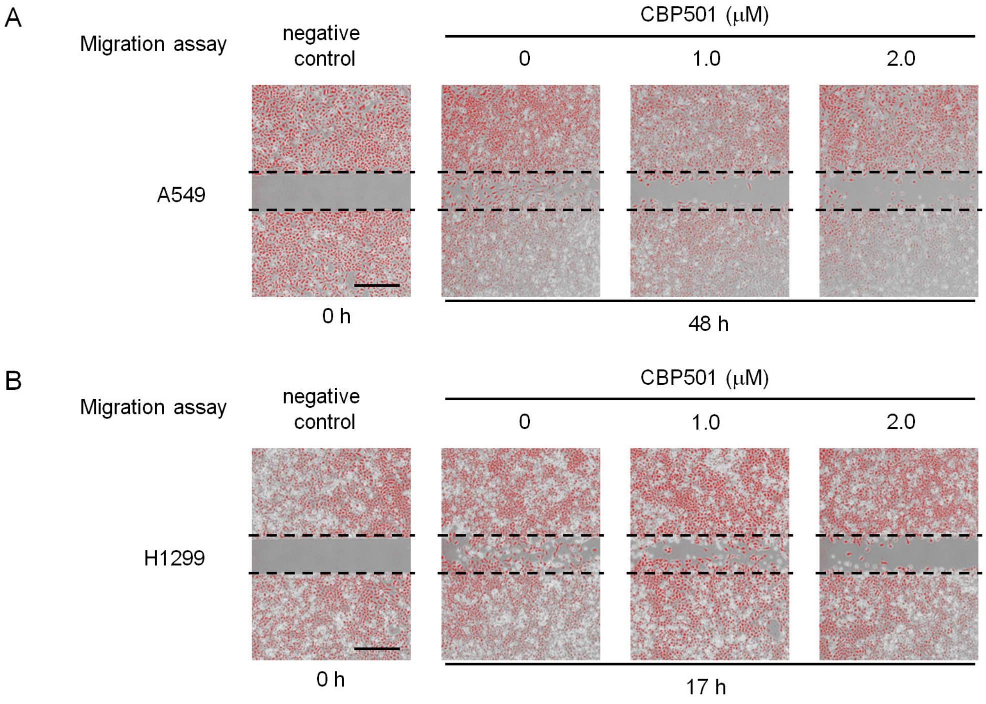

Supplementary Figure 1: CBP501 prevents cell migration using wound healing assay. A549 **(A)** and H1299 **(B)** cells were grown on an ibidi culture insert and then cells were treated with CBP501 (1 or 2 µM) for 48 or 17 h. Red signal set threshold to capture the wound closure. Scale bar is 500 µm.

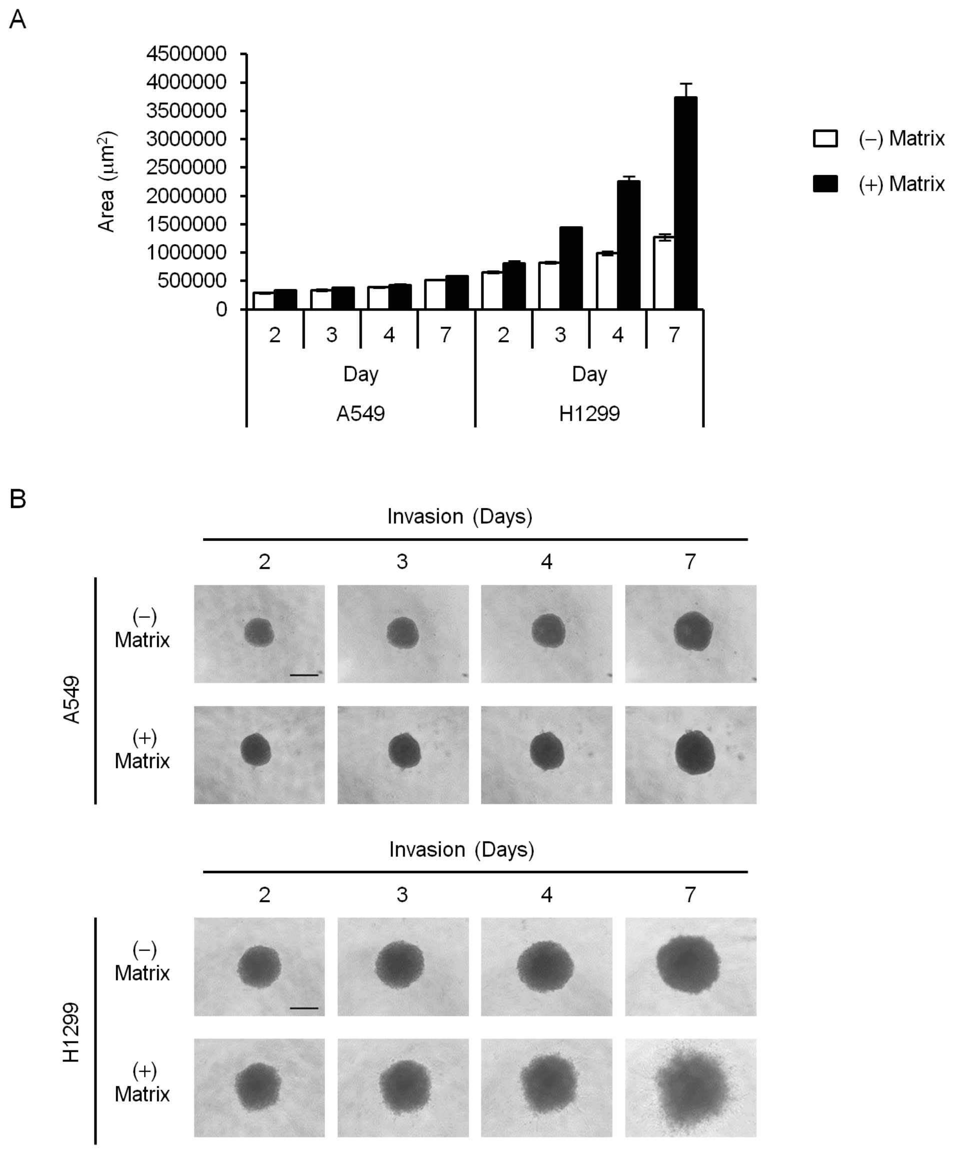

Supplementary Figure 2: Quantitative analysis of surface area for low invasive (A549) and high invasive (H1299) cell lines over a seven day period. (A) Briefly, 3×10^3 cells were resuspended in spheroid formation ECM solution and gently pelleted in a 96 well round bottom spheroid formation plate. After 3 days, spheroids were imaged. To induce invasion, the invasion matrix and medium were added to each well. Cells were allowed to invade the surrounding matrix for 7 days. **(B)** Morphology of 3-D spheroid cell invasion over a seven day period. Low invasive cells (A549) remain as cell aggregates and do not invade into the surrounding invasion matrix; whereas, high invasive cells (H1299) invade into the surrounding invasion matrix as spindle-like protrusions. Scale bar is 500 µm. Data, the mean ± SD.

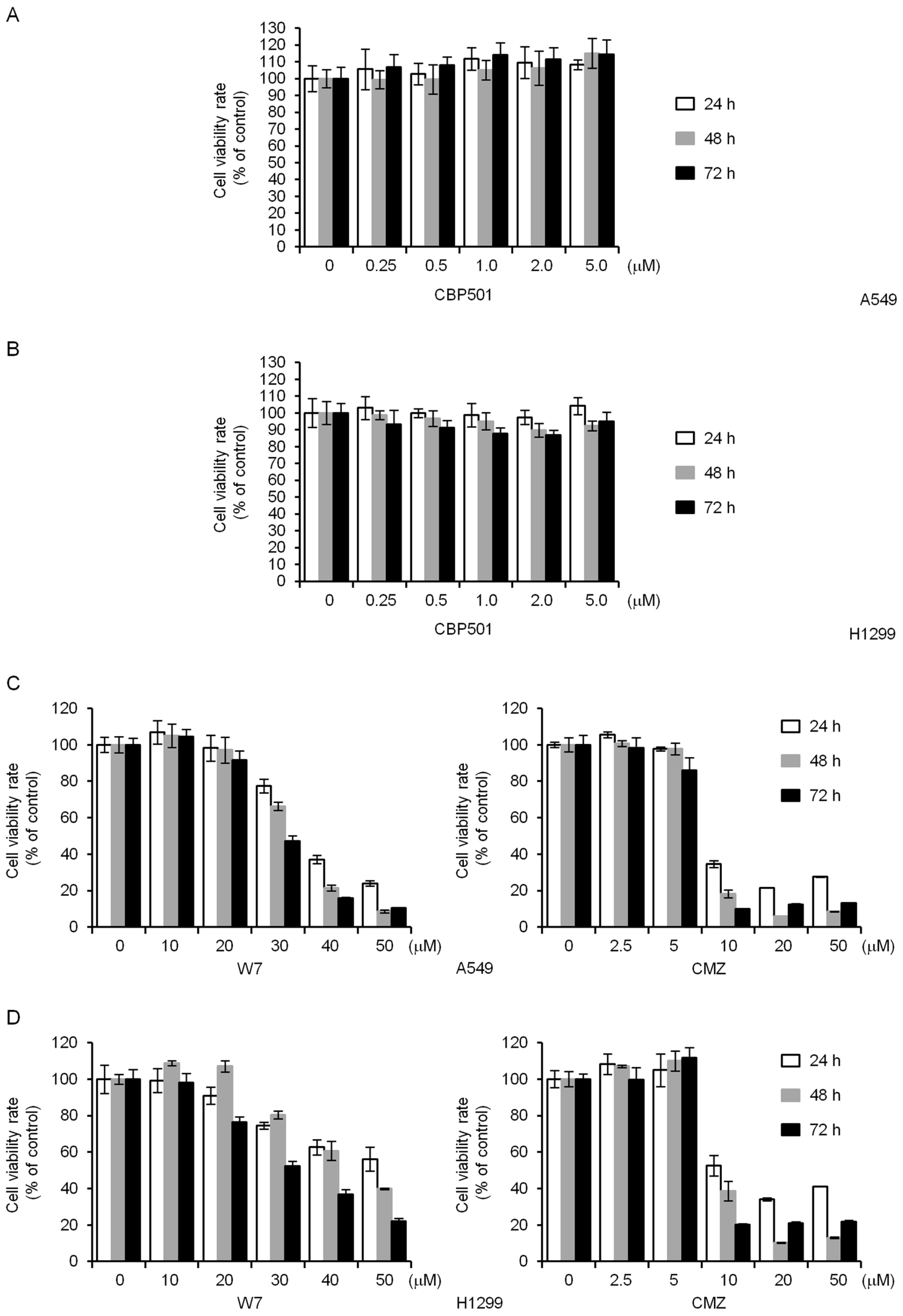

Supplementary Figure 3: Cell viability is not affected by CBP501. A549 **(A and C)** and H1299 **(B and D)** cells were treated with CBP501 (0.25-5 μM), W7 (10-50 μM) or CMZ (2.5-50 μM) for indicated period (24-72 h, *n* = 3). Cell viability was assessed at indicated times using cell counting kit-8. Data, the mean ± SD.

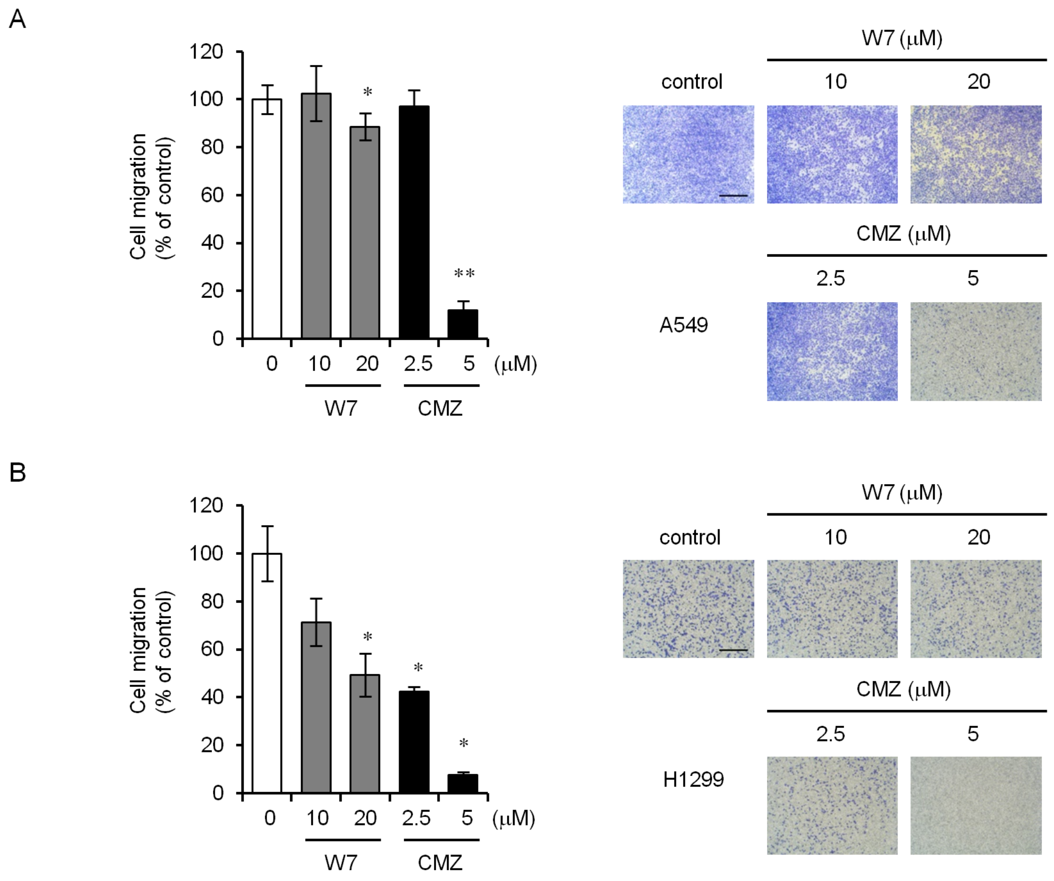

Supplementary Figure 4: W7 and CMZ prevent cell migration in A549 and H1299 cell lines. Serum-starved A549 **(A)** or H1299 **(B)** cells were treated with W7 (10 or 20 μM) or CMZ (2.5 or 5 μM) for 24 h using a transwell assay. Cell migration %ages (left) were calculated from samples eluted from the membrane ($n = 3$). Photomicrographs of the observed cell migration (right). Scale bar is 500 μm. Data, the mean ± SD; * and **, $P < 0.05$ and $P < 0.005$, respectively.

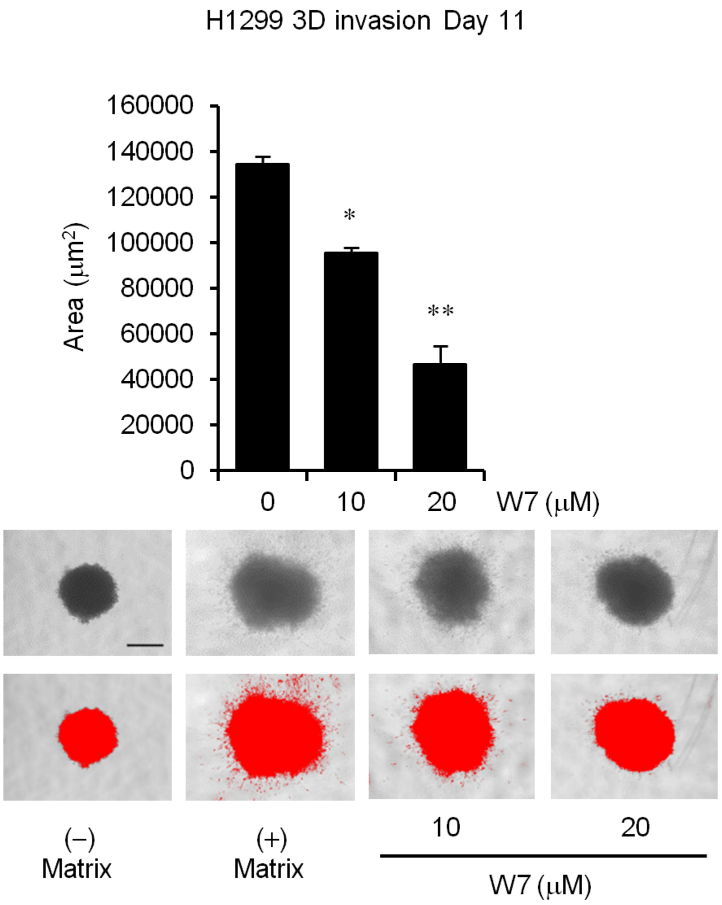

Supplementary Figure 5: W7 prevents cell invasion in H1299 cell lines. Quantification of H1299 cell invasion in spheroid invasion assay. Cells were aggregated into spheroids and then induced to invade the surrounding matrix for 11 days. The total area of invading spheroid was calculated with Image-J and considered as a measurement of cell invasion ($n = 3$). Red signal threshold was set to capture the total structure. Scale bar is 500 μm. Data, the mean ± SD; * and **, $P < 0.05$ and $P < 0.005$, respectively.

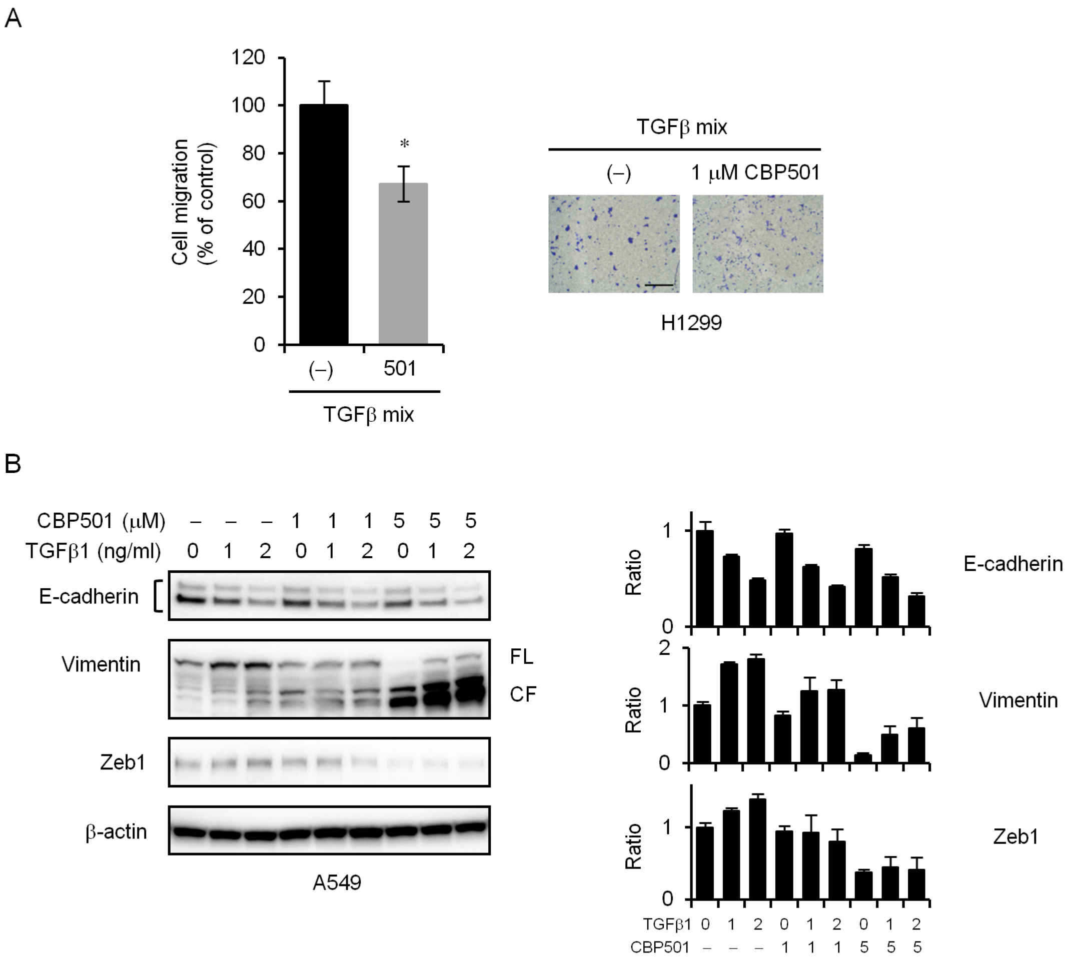

Supplementary Figure 6: CBP501 prevents cell migration and EMT of H1299 cells by TGFβ. **(A)** H1299 cells were treated during 72 h in combination with CBP501 (1 μM) with TGFβ mix (10 ng/mL TGFβ1 and 10 ng/mL TGFβ2). **(B)** A549 cells were treated with or without TGFβ1 (1 or 2 ng/mL) in combination with CBP501 (1 or 5 μM) for 72 h. Cell lysates were analyzed by western blot assay with antibodies to E-cadherin, Vimentin, Zeb1 and β-actin. The measured band density was normalized relative to that of the control sample, with the control value set to 1. β-actin was used as a loading control. The results are the average relative intensity of three replicate blots. *FL*, full length; *CF*, cleaved fragment. Scale bar is 500 μm. Data, the mean ± SD; *, $P < 0.05$.

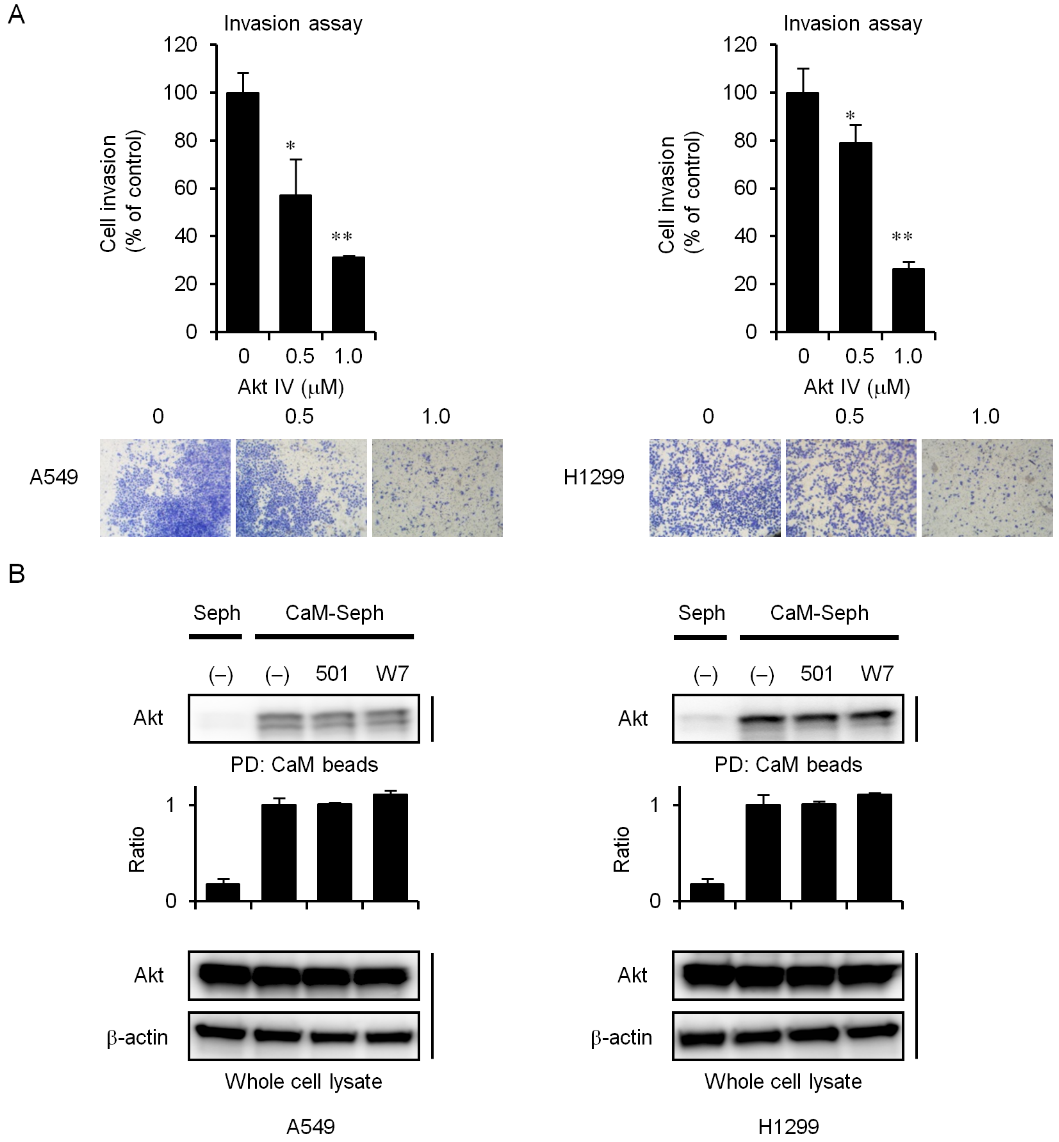

Supplementary Figure 7: CBP501 does not inhibit Akt/CaM interaction *in vitro*. (A) Serum-starved A549 or H1299 cells were treated with Akt IV (0.5 or 1 μM) for 48 h using matrigel invasion chamber. Cell invasion %ages (above) were calculated from sample eluted from the membrane (n = 3). Photomicrographs of the observed cell migration (below). **(B)** Cellular lysates (1 mL) were incubated with CaM-sepharose (CaM-seph) or Plain (Seph) beads with CBP501 (2 μM), W7 (20 μM) in the presence of Ca^{2+} as indicated in Materials and Methods. Cell lysates were analyzed by western blot assay with antibodies to Akt and β-actin (n=3).
